# Early-Stage Lung Cancer Treatment Disparities by Race Among Medicare Beneficiaries

**DOI:** 10.1001/jamanetworkopen.2025.59845

**Published:** 2026-03-02

**Authors:** Olivia F. Lynch, Do H. Lee, Pamela R. Soulos, James B. Yu, Jeph Herrin, Cary P. Gross

**Affiliations:** 1 National Clinician Scholars Program, Yale School of Medicine, Yale University, New Haven, Connecticut; 2 Cancer Outcomes, Public Policy and Effectiveness Research (COPPER) Center, Yale School of Medicine, Yale University, New Haven, Connecticut; 3 Department of Radiation Oncology, Dartmouth-Hitchcock Medical Center, Lebanon, New Hampshire

## Abstract

**Importance:**

Prior work demonstrated racial disparities in lung cancer care for Medicare beneficiaries in the 1990s and early 2000s, with a 13% to 15% difference in receipt of curative therapy between Black and White patients. With the evolving landscape of treatment modalities and the addition of stereotactic body radiotherapy (SBRT) as the standard of care, it is unknown whether disparities have persisted.

**Objective:**

To assess temporal trends in racial disparities in the receipt of curative treatment, as well as specific curative modalities, for early-stage NSCLC among Medicare beneficiaries.

**Design, Setting, and Participants:**

This retrospective cohort study used the Surveillance, Epidemiology, and End Results (SEER)–Medicare linkage to identify 28 287 non-Hispanic Black and non-Hispanic White patients aged 66 to 85 years who received a diagnosis of stage I or II NSCLC in 2005 to 2007, 2011 to 2013, and 2017 to 2019. Statistical analysis was performed from November 2024 to April 2025.

**Exposure:**

Race (non-Hispanic Black compared with non-Hispanic White), as defined in the SEER-Medicare data.

**Main Outcomes and Measures:**

Outcomes included receipt of any curative treatment (surgery or radiotherapy) for all patients, receipt of surgery or radiotherapy, and receipt of preferred treatment (lobectomy or SBRT) among patients receiving surgery or radiotherapy, respectively. Disparities in the data were compared with findings from the 1990s and early 2000s.

**Results:**

Among 28 287 patients (mean [SD] age, 75.1 [5.3] years; 52.4% men; 7.5% Black and 92.5% White), 82.3% (95% CI, 81.8%-82.7%) received curative treatment. Adjusted probabilities of receiving curative treatment were significantly lower for Black compared with White patients in each period: 73.9% (95% CI, 70.6%-77.1%) of Black patients and 83.3% (95% CI, 82.5%-84.1%) of White patients from 2005 to 2007 (difference, –9.4% [95% CI, −12.8% to −6.1%]; *P* < .001); 76.3% (95% CI, 73.2%-79.4%) of Black patients and 85.2% (95% CI, 84.4%-86.0%) of White patients from 2011 to 2013 (difference, –8.9% [95% CI, −12.1% to −5.8%]; *P* < .001); and 78.4% (95% CI, 75.3%-81.4%) of Black patients and 86.8% (95% CI, 86.0%-87.5%) of White patients from 2017 to 2019 (difference, –8.4% [95% CI, −11.6% to −5.3%]; *P* < .001). Disparities were associated primarily with differences in surgical treatment, with 52.3% of Black patients compared with 65.9% of White patients undergoing surgery from 2005 to 2007, 48.3% of Black patients compared with 61.0% of White patients undergoing surgery from 2011 to 2013, and 43.7% of Black patients compared with 53.1% of White patients undergoing surgery from 2015 to 2017 (*P* < .001 for all). SBRT use increased sharply, and disparities in its use emerged only during the time interval of its initial implementation, from 2011 to 2013 (Black, 39.6%; White, 51.6%; *P* = .004).

**Conclusions and Relevance:**

This study suggests that racial disparities in the receipt of curative treatment for early-stage NSCLC among Medicare beneficiaries have persisted since the 1990s, with the most marked differences in the receipt of surgical treatment and significant racial differences in the receipt of SBRT as the therapy emerged. Further work must explore factors that can address and mitigate these disparities.

## Key Points

**Question:**

Have racial disparities in the receipt of curative treatment for early-stage non–small cell lung cancer (NSCLC) among Medicare beneficiaries changed compared with patterns documented in the 1990s?

**Findings:**

In this cohort study using SEER-Medicare data from 2005 to 2019, Black patients had significantly lower adjusted rates of curative therapy than White patients. Lower rates of curative therapy among Black patients were associated with persistently lower receipt of surgery, while overall rates of receipt of radiotherapy were not consistently different by race.

**Meaning:**

This study suggests that disparities between Black and White patients in curative treatment persist for Medicare beneficiaries with early-stage NSCLC, highlighting structural barriers to certain cancer treatments.

## Teaser

This cohort study uses Surveillance, Epidemiology, and End Results–Medicare data to assess recent trends in racial disparities in the receipt of curative treatment, as well as specific curative modalities, for early-stage non–small cell lung cancer among Medicare beneficiaries.

## Introduction

Substantial racial and ethnic disparities exist in the receipt of curative therapy for early-stage non–small cell lung cancer (NSCLC).^1,2,3,4,5^ Lower rates of lung cancer surgery for Black patients compared with White patients were first identified in the early 1990s within the Medicare population—a group that is insured and ostensibly should have uniform access to care compared with the general US population.^6^ Subsequent work found that these racial disparities in the treatment of Medicare beneficiaries with NSCLC did not change between the early 1990s and the early 2000s, despite the substantial attention devoted to this issue.^1^ Other work further validates the finding of continued racial disparities in surgical treatment of early-stage NSCLC.^3,4,5^

Over the past 20 years, however, the approach to curative therapy for patients with early-stage NSCLC has changed, and treatment options now include both surgery and radiotherapy. Although prior analyses suggest that surgical resection yields improved survival compared with radiotherapy in general, radiotherapy is now seen as a viable and efficacious alternative to surgery for select patients.^7^ The choice of specific modalities (ie, surgery vs radiotherapy) and the within-modality treatment patterns (ie, the type of radiotherapy or type of surgery) could each be affected by patient health status, clinician bias, or health system inequity. A recent study of the Medicare population found that Black patients had lower odds of receiving stage-appropriate evaluation and treatment.^8^

Approaches for radiotherapy or surgical resection have also evolved considerably. Stereotactic body radiotherapy (SBRT)—a type of radiotherapy—originated in the 1990s at the Karolinska Institute in Sweden and came into broader clinical use in the US in the early 2000s, with its use increasing from 0.3% of patients with stage 1 NSCLC in 2002 to approximately 9% of patients in 2011.^9^ Since then, SBRT has become the predominant radiotherapy modality among patients undergoing curative therapy for early-stage lung cancer, given that SBRT is associated with better outcomes than traditional conventionally fractionated radiotherapy. In comparison with surgery, SBRT has been shown to have lower rates of immediate mortality and toxic effects.^10^ The National Comprehensive Cancer Network (NCCN) treatment guidelines have incorporated SBRT as an appropriate alternative to surgery for patients with early-stage disease since 2015, with conventionally fractionated 3-dimensional conformal radiotherapy considered the less preferred alternative.^11^ Similarly, surgical approaches have evolved. Sublobar resections such as segmentectomy and wedge have gained popularity for patients with greater comorbidity; however, lobectomy is still generally associated with superior cancer outcomes.^12,13,14^

Given the rapidly evolving clinical landscape, it is important to assess trends in treatment of early-stage NSCLC through an equity lens. We therefore examined recent trends in racial disparities in the receipt of curative treatment for early-stage NSCLC and whether they had improved compared with disparities initially documented in the 1990s. We also evaluated racial disparities over time in the receipt of specific treatment modalities (surgery or radiotherapy) and in the receipt of preferred treatment (lobectomy or SBRT) to assess whether more novel, cutting-edge therapies equitably reached patients regardless of race. We expected that the receipt of radiotherapy would exhibit the greatest disparity in the beginning of our study period and would have improved over time as it became a more widespread option and that the magnitude of disparities in the receipt of surgery would remain stable. We assessed these aims among Medicare fee-for-service beneficiaries who received a diagnosis of early-stage NSCLC during 3 time periods (2005-2007, 2010-2012, and 2017-2019) and compared the findings with those of prior work, which focused on patients who received a diagnosis in 1992 to 2002.

## Methods

### Study Design and Patient Sample

Using the Surveillance, Epidemiology, and End Results (SEER)–Medicare data linkage, we included beneficiaries who received a diagnosis of stage I or II NSCLC during the 3 time periods (2005-2007, 2011-2013, and 2017-2019) to evaluate temporal trends. We restricted our analysis to non-Hispanic Black (hereafter, *Black*) and non-Hispanic White (hereafter, *White*) patients who were 66 to 85 years of age at diagnosis with histologic findings consistent with NSCLC (eFigure in Supplement 1). Race and ethnicity were identified using a combination of Medicare’s Research Triangle Institute race variable and SEER-provided ethnicity information, abstracted from medical records. Patients must have had continuous Medicare fee-for-service and Part B coverage from 1 year prior to diagnosis through 6 months after lung cancer diagnosis with at least 1 claim billed to Medicare during 1 year prior to 6 months after diagnosis (eTable 1 in Supplement 1). We included all 17 SEER regions.^15^ The prior 1992 to 2002 study^1^ included only the 9 SEER regions in existence at that time—San Francisco, Connecticut, Detroit, Hawaii, Iowa, New Mexico, Seattle, Utah, and Atlanta (eTable 2 in Supplement 1). Accordingly, we conducted a sensitivity analysis that focused only on residents of these regions. The institutional review board at Yale University determined that this study does not meet the definition of human participants research and waived the requirement for institutional review board review. The data presented here are reported in accordance with the Strengthening the Reporting of Observational Studies in Epidemiology (STROBE) reporting guideline for cohort studies, with structured reporting of the cohort, exposures, outcomes, covariates, statistical methods, and results to ensure transparency and reproducibility.

### Outcomes

Our outcomes were the receipt of any curative treatment, receipt of surgery or radiotherapy, and receipt of any preferred treatment. *Curative treatment*, which was defined as the standard-of-care therapy based on NCCN guidelines, included surgery or radiotherapy. *Surgery* included patients undergoing lobectomy, sublobar resection, pneumonectomy, or other surgeries, such as wedge resection or sleeve resection. *Radiotherapy* included patients receiving conventional radiotherapy, SBRT, and proton therapy. *Preferred treatment* was defined as lobectomy or SBRT. This was assigned because according to the 2019 NCCN guidelines,^16^ anatomic resection was preferred for most patients with NSCLC. For SBRT, according to the 2019 NCCN guidelines, patients with T2 or T1 N0 disease are recommended to receive SBRT, which aligns with stage I or IIA. Some patients may have lesions too centrally located to be ideal SBRT candidates; however, we do not believe this factor would vary between the racial subgroups we are comparing. Patients receiving proton therapy were excluded from this preferred treatment analysis because we cannot categorize this treatment as preferred vs nonpreferred. If patients had treatment codes for both SBRT and conventional radiotherapy, they were included in the SBRT (preferred) group. SBRT was limited to 5 fractions, and patients were excluded from this subanalysis if they received more than 5 SBRT fractions, as that may represent metastatic disease, multiple primary neoplasms, or a coding error. For our analysis of treatment modality (surgery vs radiotherapy), patients were assigned to the surgery subgroup if they received both radiotherapy and surgery, as surgery is the more definitive therapy.

### Statistical Analysis

Statistical analysis was performed from November 2024 to April 2025. We summarized all covariates by race, reporting standardized mean differences (SMDs) for each. We also summarized crude outcome rates by racial group. To assess disparities in each time period and changes over time, we estimated a series of multivariate logistic regression models. Candidate covariates were selected based on clinical expertise and were consistent with the published literature.^17,18^ To compare results with prior work, we included similar covariates as a 2008 study.^1^ For each outcome, we ran a series of bivariate regressions of all candidate covariates and retained variables for which *P* < .20, all of which were logically relevant to our research question and similar to those used in other work on treatment disparities. Covariates included sociodemographic variables (age, sex, diagnosis year, marital status, SEER region, and urban or rural residence) and clinical variables including the Elixhauser comorbidity index^19,20^ and frailty,^21^ which used the Kim Frailty Index—a validated, claims-based measurement of a patient’s frailty status.^21,22^ We also considered 3 measures of access to care—primary care clinician visit, hospitalization in the year prior to cancer diagnosis, and receipt of the influenza vaccine in the year prior to diagnosis.

Next, to assess whether changes in the receipt of therapy over time differed by race, we estimated a single model for each outcome, including time period, race, a time × race interaction term, and all covariates retained in bivariate analyses. Finally, we ran stratified analyses by time period to estimate the adjusted probability of receiving any treatment, surgery, radiotherapy, or preferred treatment for Black compared with White patients within each time period. To more directly compare results with the 2008 study,^1^ we performed sensitivity analyses in which we replicated the main analysis, restricting our sample to only patients from the 9 SEER regions and including only covariates that were included in the 2008 study.

For each model, we report the estimated probabilities, *P* value, and the marginal difference in outcome rates between groups at each time period. All *P* values were from 2-sided tests and results were deemed statistically significant at *P* < .05. All analyses were performed using Stata MP, version 18.0 (StataCorp LLC),^23^ SAS, version 9.4 (SAS Institute Inc), and R, version 4.3.1 (R Project for Statistical Computing).

## Results

A total of 28 287 patients (mean [SD] age, 75.1 [5.3] years; 52.4% men and 47.6% women; 7.5% Black and 92.5% White) were included in our study (Table 1). Black and White patients were similar across most demographic characteristics; marital status was the most notably different, with 53.5% of White patients married compared with 33.2% of Black patients (SMD, 0.42). In terms of clinical characteristics and care access, Black patients were more likely to have 3 or more comorbidities (Black, 37.7%; White, 28.6%; SMD, 0.20), to meet criteria for frailty (moderately or severely frail: Black, 12.3%; White, 7.2%; SMD, 0.23), and to have been hospitalized in the year prior to diagnosis (Black, 33.3%; White, 28.0%; SMD, 0.11). Black patients were less likely to have received an influenza vaccine in the year prior to diagnosis (Black, 41.0%; White, 59.3%; SMD, 0.37).

**Table 1.** Characteristics of the Study Population

Characteristic	No. (%)		SMD
	Black patients (n = 2117)	White patients (n = 26 170)	
Age, mean (SD), y	74.3 (5.1)	75.1 (5.1)	0.16
Sex			
Female	1030 (48.7)	12 444 (47.6)	0.02
Male	1087 (51.3)	13 726 (52.4)	
Marital status (married)	702 (33.2)	14 012 (53.5)	0.42
Urban residence	2093 (98.9)	25 490 (97.4)	0.11
Time of diagnosis			
Early (2005-2007)	718 (33.9)	9113 (34.8)	0.04
Middle (2011-2013)	726 (34.3)	8547 (32.7)	
Late (2017-2019)	673 (31.8)	8510 (32.5)	
Elixhauser Comorbidity Index			
0	527 (24.9)	7267 (27.8)	0.20
1-2	792 (37.4)	11 418 (43.6)	
≥3	798 (37.7)	7485 (28.6)	
Kim Frailty Index			
Not frail	46 (2.2)	1003 (3.8)	0.23
Prefrail	1145 (54.1)	15 941 (60.9)	
Mildly frail	666 (31.5)	7339 (28.0)	
Moderately frail	195 (9.2)	1555 (5.9)	
Severely frail	65 (3.1)	332 (1.3)	
Primary care clinician visit in year prior to diagnosis	1560 (73.7)	20 803 (79.5)	0.14
Hospital admission in year prior to diagnosis	704 (33.3)	7332 (28.0)	0.11
Receipt of influenza vaccine in year prior to diagnosis	868 (41.0)	15 511 (59.3)	0.37

Abbreviation: SMD, standardized mean difference.

A total of 82.3% (95% CI, 81.8%-82.7%) of patients received curative treatment. Across the 3 time intervals, the crude receipt of any curative treatment ranged from 68.2% to 72.2% for Black patients and from 82.0% to 84.9% for White patients (Table 2). Black patients experienced a 13.8% lower percentage of curative therapy than White patients in 2005 to 2007, a 13.7% lower percentage of curative therapy than White patients in 2011 to 2013, and a 12.7% lower percentage of curative therapy than White patients in 2017 to 2019.

**Table 2.** Receipt of Curative Treatment by Race and Time Period, Crude and Adjusted

Patient group	Earlier publication^a^		Current study^b^		
	1992-1994	2000-2002	2005-2007	2011-2013	2017-2019
Crude, %					
Black	68.6	59.8	68.2	69.3	72.2
White	81.9	75.3	82.0	83.0	84.9
Black-White difference	−13.3	−15.5	−13.8	−13.7	−12.7
Adjusted, % (95% CI)					
Black	73.1 (72.3 to 73.9)	64.9 (64.0 to 65.8)	73.9 (70.6 to 77.1)	76.3 (73.2 to 79.4)	78.4 (75.3 to 81.4)
White	84.9 (84.2 to 85.6)	79.3 (78.5 to 80.1)	83.3 (82.5 to 84.1)	85.2 (84.4 to 86.0)	86.8 (86.0 to 87.5)
Black-White difference	−11.8 (−12.9 to −10.7)	−14.4 (−15.6 to −13.2)	−9.4 (−12.8 to −6.1)	−8.9 (−12.1 to −5.8)	−8.4 (−11.6 to −5.3)
Difference in Black-White difference	NA	NA	Reference	0.5 (−4.0 to 5.0)	1.0 (−3.5 to 5.5)

Abbreviation: NA, not applicable.

^a^ Adjusted for age, sex, marital status, physician visits, geographic region, cancer stage, and comorbid conditions.

^b^ Adjusted for race; age; sex; marital status; urban residence; Surveillance, Epidemiology, and End Results region; cancer stage; time of diagnosis; Elixhauser Comorbidity Index; Kim Frailty Index; primary care clinician visit in the year prior to diagnosis; hospital admission in year prior to diagnosis; and receipt of influenza vaccine in year prior to diagnosis.

After adjustment for demographic and clinical characteristics, there was a significant difference in the receipt of curative treatment throughout all intervals of our study (Table 2). From 2005 to 2007, the estimated probability of Black patients receiving curative treatment was 73.9% (95% CI, 70.6%-77.1%) compared with 83.3% (95% CI, 82.5%-84.1%) of White patients (difference, –9.4% [95% CI, −12.8% to −6.1%]; *P* < .001). From 2011 to 2013, 76.3% (95% CI, 73.2%-79.4%) of Black patients compared with 85.2% (95% CI, 84.4%-86.0%) of White patients received curative treatment (difference, –8.9% [95% CI, −12.1% to −5.8%]; *P* < .001), and from 2017 to 2019, 78.4% (95% CI, 75.3%-81.4%) of Black patients compared with 86.8% (95% CI, 86.0%-87.5%) of White patients received curative treatment (difference, –8.4% [95% CI, −11.6% to −5.3%]; *P* < .001) (Table 2). The time × race interaction term included in our adjusted model was not statistically significant (*P* = .65). In the sensitivity analysis restricted to the original SEER regions and covariates, results were similar.

**Figure 1.**
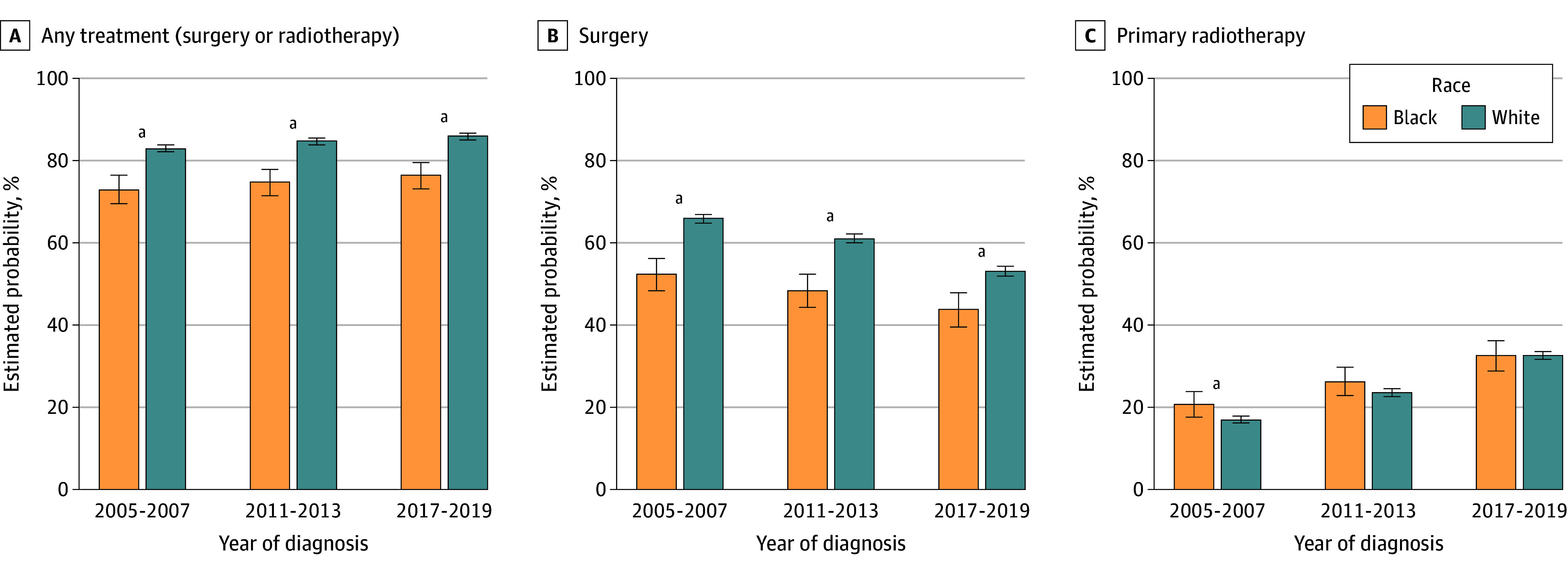
Bar Graph of Estimated Probability of Receipt of Curative Therapy by Treatment Modality Adjusted for race; age; sex; marital status; urban residence; Surveillance, Epidemiology, and End Result region; cancer stage; time of diagnosis; Elixhauser Comorbidity Index; Kim Frailty Index; primary care clinician visit in year prior to diagnosis; hospital admission in year prior to diagnosis; and receipt of influenza vaccine in year prior to diagnosis. Error bars indicate 95% CIs. ^a^Statistically significant difference between Black and White patients at *P* < .05.

The association between race and receipt of treatment differed by treatment modality. Disparities in the receipt of surgery persisted across time. From 2005 to 2007, the probability of Black patients undergoing surgery was 52.3% (95% CI, 48.2%-56.2%) compared with 65.9% (95% CI, 64.8%-66.9%) of White patients (difference, −13.6%; 95% CI, −17.7% to −9.5%; *P* < .001) (Figure 1). In the 2 subsequent time intervals, the receipt of surgery decreased for all patients, but disparities persisted: from 2011 to 2013, the probability of Black patients receiving surgery was 48.3% (95% CI, 44.3%-52.3%) compared with 61.0% (95% CI, 59.9%-62.1%) for White patients, and from 2017 to 2019, the probability of Black patients receiving surgery was 43.7% (95% CI, 39.5%-47.8%) compared with 53.1% (95% CI, 52.0%-54.3%) for White patients, all statistically significant. In contrast, the estimated probability of receiving radiotherapy increased for all patients as time elapsed: 17.0% (95% CI, 16.2%-17.8%) of Black patients and 20.7% (95% CI, 17.6%-23.8%) of White patients from 2005 to 2007 and 32.6% (95% CI, 28.9%-36.3%) of Black patients and 32.6% (95% CI, 31.6%-33.7%) of White patients from 2017 to 2019.

**Figure 2.**
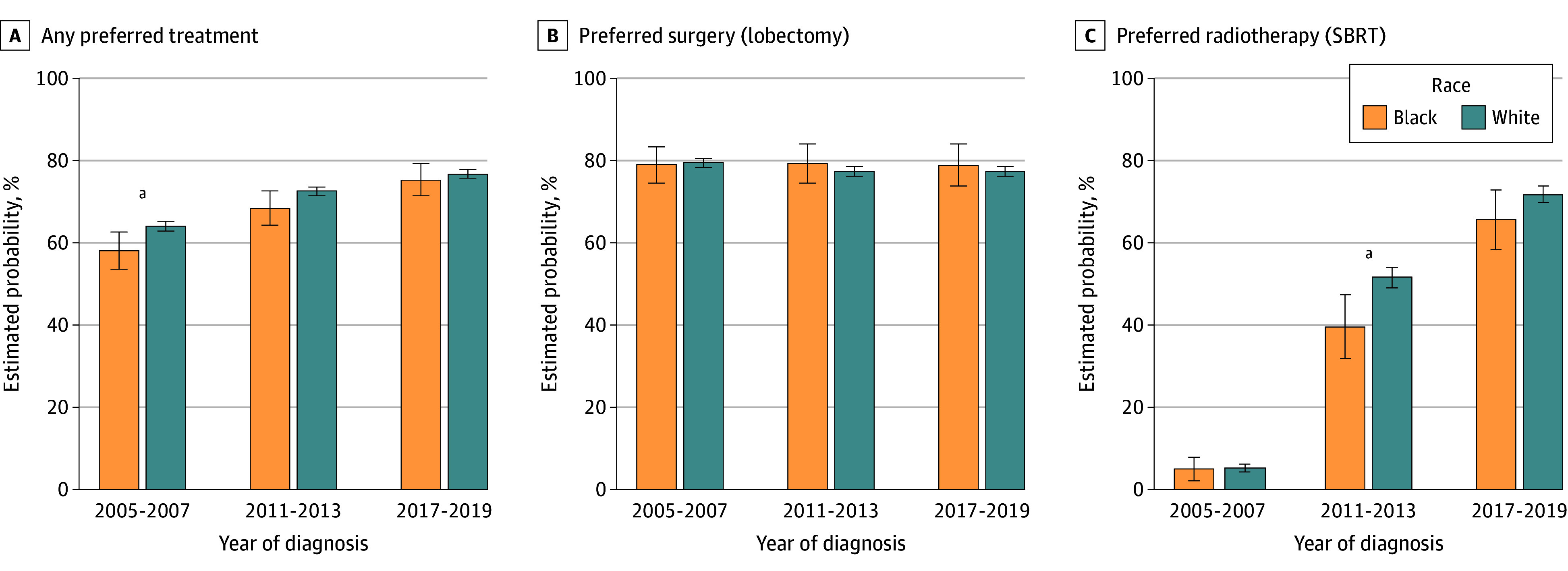
Bar Graph of Estimated Probability of Receipt of Preferred Treatment by Treatment Modality Adjusted for race; age; sex; marital status; urban residence; Surveillance, Epidemiology, and End Result region; cancer stage; time of diagnosis; Elixhauser Comorbidity Index; Kim Frailty Index; primary care clinician visit in year prior to diagnosis; hospital admission in year prior to diagnosis; and receipt of influenza vaccine in year prior to diagnosis. Error bars indicate 95% CIs. SBRT indicates stereotactic body radiotherapy. ^a^Statistically significant difference between Black and White patients at *P* < .05.

Among patients who received treatment, disparities in the receipt of each preferred treatment modality (SBRT or lobectomy) were less marked. The estimated probability of patients undergoing radiotherapy receiving SBRT was 5.1% (95% CI, 2.2%-8.0%) for Black patients and 5.3% (95% CI, 4.4%-6.2%) for White patients from 2005 to 2007; however, as the therapy gained popularity, disparities widened, with 39.6% (95% CI, 31.9%-47.4%) of Black patients receiving SBRT from 2011 to 2013 compared with 51.6% (95% CI, 49.1%-54.0%) of White patients (*P* = .004; difference-in-difference, −11.8%; 95% CI, −20.3% to −3.1%; *P* = .008) (Figure 2). From 2017 to 2019, however, this difference had narrowed and was no longer statistically significant, with 65.6% (95% CI, 58.4%-72.8%) of Black patients receiving SBRT compared with 71.7% (95% CI, 69.8%-73.7%) of White patients (difference, −6.1%; 95% CI, −13.6% to 1.4%; *P* = .11). The receipt of preferred surgery for surgical patients was similar between Black and White patients, with no significant differences in any of the 3 time periods.

Although our time × race interaction term was not statistically significant in any of our models, we further analyzed the degree to which disparities fluctuated over time by calculating Black-White estimated probability differences for each year group. As evidenced by significantly overlapping 95% CIs, these patterns do not indicate a statistically significant widening or narrowing of disparities, supporting the interpretation of a largely persistent gap over time.

## Discussion

Our study found persistent racial disparities in the receipt of curative treatment for Medicare beneficiaries with early-stage NSCLC compared with data from the 1990s to early 2000s. These findings build on prior work in several important ways. First, by using the same data source and methodology as the earlier study, and accounting for clinical and sociodemographic characteristics, we found that disparities persisted in this population that had similar insurance.^1^ Second, we found marked differences in the approach to curative treatment—while surgical disparities persisted, we did not find consistent racial disparities in the receipt of radiotherapy.

The persistence of disparities is particularly concerning given the substantial attention devoted to rectifying these differences over time. Several national public health initiatives implemented in the early 2000s, as well as work done by advocacy organizations, attempted to address racial disparities in lung cancer treatment. This included the establishment of the Center to Reduce Cancer Health Disparities (CRCHD) in 2001 by the National Cancer Institute,^24^ patient navigator programs aimed at guiding patients from underserved and racial and ethnic minority populations through the cancer treatment process, and patient-focused educational initiatives.^25,26,27^ Despite this work, this study demonstrates that over a 30-year time period, racial differences in the receipt of treatment for lung cancer have narrowed only approximately 4%.

There has been a substantive change in the use of curative treatment modalities over time, with disparities largely associated with differences in the receipt of surgery, rather than radiotherapy. Our study found a persistently lower probability of receipt of surgery for Black patients compared with White patients, as well as an overall reduction in surgery over time for all patients. As fewer invasive modalities such as SBRT have become validated first-line treatments with comparable outcomes, fewer patients and clinicians may be opting for surgery.^7^ In addition, for patients with multiple comorbidities, radiotherapy can be a preferable and less invasive treatment option; the increasing prevalence of chronic disease may therefore be shifting the population away from surgery.^28^ When it comes to racial disparities, although we adjusted for frailty and comorbidity, there may be racial differences in health status that we still are not accounting for in our model. Alternatively, studies have shown that a lower supply of surgeons in a given catchment area reduces access to surgical services.^29,30^ Thus, there may be higher barriers to accessing surgeons in our population that do not apply to radiotherapy oncologists, leading to the disparity patterns we observed.

We did not observe disparities in the receipt of overall radiotherapy over time. Radiotherapy treatments do not require hospitalization or postoperative care, as surgery does. This relative convenience and outpatient delivery may have been associated with the modality being more accessible to patients with limited transportation or social support, particularly as the technology matured. Still, some studies have found that for patients with fewer comorbidities, surgery is associated with better outcomes compared with SBRT; thus, tailoring treatment plans to specific patients and preserving access to surgery for patients who are eligible are critical.^31^

When looking at preferred treatment modalities, we found significant racial disparities in the receipt of SBRT during the time when it was being rapidly adopted into clinical practice, but no disparities in the receipt of lobectomy. After SBRT was incorporated into the NCCN guidelines for early-stage lung cancer in the early 2010s, we found an 8-fold increase in the percentage of patients who underwent radiotherapy receiving SBRT.^32^ However, this adoption was inequitable; in the 2011 to 2013 time period, only 39.6% of Black patients received SBRT compared with 51.6% of White patients. By 2017 to 2019, this disparity was no longer significant. This finding is consistent with prior studies, which have demonstrated that newer treatment modalities—from glucagon-like peptide-1 agonists to multiple myeloma treatments—often have larger racial disparities when initially implemented.^9,31,33,34,35^ In contrast, the percentage of surgical patients receiving lobectomy—a longstanding standard-of-care treatment—was relatively unchanged over time and equivalent for Black and White patients, suggesting that while there may be barriers to accessing surgery, patients who do undergo surgery are receiving the preferred treatment regardless of race. There are some clinical scenarios in which nonanatomic resections, including wedge and sublobar resection, might be considered equivalently preferred to lobectomy; however, according to the NCCN guidelines at the time of our study,^16^ anatomic resection was preferred for most patients, consistent with our study rationale.

### Limitations

Our study has some limitations. First, we restricted our patient population to those 65 years or older with Medicare fee-for-service insurance. Many public health initiatives in the past 20 years have attempted to target lung cancer screening and treatment access in younger patient populations, including lowering the age and minimum smoking history that qualifies for screening; thus, we may not be appreciating the effect of such programs with our study.^36^ Second, by restricting our population to insured patients, we may be underestimating the true racial disparities in receipt of treatment.^37^ In addition, assessment of comorbidity and frailty is limited with administrative claims data and inadequately captures the highly individualized patient-level decision-making that factors into treatment plans. Studies have shown a higher prevalence of frailty among Black patients with cancer compared with White patients with cancer.^38^ This difference may partially mediate the disparate outcomes in the receipt of treatment that we observed in our study. As well, definitions and indices of frailty and comorbidity are not without their own biases, and misclassification of Black patients as more or less frail or comorbid compared with White patients may be associated with Black patients receiving less treatment upfront and/or may mute even greater disparities in the receipt of treatment in our analyses.^39^ Third, treatment decisions are highly nuanced and personalized, involving factors not able to be captured in our data. A patient’s financial situation, level of social support, and cultural beliefs are critical components of shared decision-making with clinicians and may well be significantly associated with the treatment patterns we observed.

## Conclusions

In this cohort study of Medicare beneficiaries who received a diagnosis of early-stage NSCLC from 2005 to 2019, we found persistent racial disparities in the receipt of curative treatment, with little improvement over the past 30 years, despite numerous public and private initiatives. As treatment evolves and new, better treatments such as SBRT have emerged, Black patients still have not been granted access to such treatment to the same degree as White patients. Even surgery—the most longstanding treatment modality for this disease—has continued to show differences in uptake that have not changed over time, reinforcing known barriers to surgical care for racial and ethinic minority populations.^8,40^ Further work must explore factors that can address and mitigate these disparities.

## Notes

Supplement 1. **eTable 1.** Codes Used to Define Radiation Treatments and Surgical Procedures **eTable 2.** SEER Regions – 2008 Study vs Our Study **eFigure.** Study Selection Criteria

Supplement 2.
Data Sharing Statement
