# Supplementary material for: Early-Stage Lung Cancer Treatment Disparities by Race Among Medicare Beneficiaries
Source: JAMA Netw Open. 2026 Mar 2;9(3):e2559845. doi: 10.1001/jamanetworkopen.2025.59845 (PMC12954539; doi:10.1001/jamanetworkopen.2025.59845)
Supplement: Supplement 1. — eTable 1. Codes Used to Define Radiation Treatments and Surgical Procedures eTable 2. SEER Regions – 2008 Study vs Our Study eFigure. Study Selection Criteria [file jamanetwopen-e2559845-s001.pdf]

Supplementary Online Content

Lynch OF, Lee DH, Soulos PR, Yu JB, Herrin J, Gross CP. Early-stage lung cancer treatment disparities by race among Medicare beneficiaries. *JAMA Netw Open.* 2026;9(3):e2559845. doi:10.1001/jamanetworkopen.2025.59845

eTable 1. Codes Used to Define Radiation Treatments and Surgical Procedures
eTable 2. SEER Regions – 2008 Study vs Our Study
eFigure. Study Selection Criteria

This supplementary material has been provided by the authors to give readers additional information about their work.

© 2026 Lynch OF et al. *JAMA Network Open.*

eTable 1. Codes Used to Define Radiation Treatments and Surgical Procedures

Treatment Type	Coding System	Codes Used
**Radiation**		
**Stereotactic Beam Radiotherapy (SBRT)**	CPT/HCPCS Codes	77373, 61793[a], 0082T[b], 0083T[b], G0173[c], G0243[b], G0251[c], G0339, G0340
	ICD-9 Procedure Codes	92.30, 92.31, 92.32, 92.33, 92.39
	ICD-10 Procedure Codes	DB20DZZ, DB20HZZ, DB20JZZ, DB21DZZ, DB21HZZ, DB21JZZ, DB22DZZ, DB22HZZ, DB22JZZ, DB25DZZ, DB25HZZ, DB25JZZ, DB26DZZ, DB26HZZ, DB26JZZ, DB27DZZ, DB27HZZ, DB27JZZ, DB28DZZ, DB28HZZ, DB28JZZ
**Non-SBRT Radiation**	CPT/HCPCS Codes	77401, 77402, 77403[c], 77404[c], 77406[c], 77407, 77408[c], 77409[c], 77411[c], 77412, 77413[c], 77414[c], 77416[c], G6003, G6004, G6005, G6006, G6007, G6008, G6009, G6010, G6011, G6012, G6013, G6014, 77301, 77385, 77386, 77418[c], 0073T[c], G6015, G6016
	ICD-9 Procedure Codes	92.2, 92.24, 92.25, 92.26
	ICD-10 Procedure Codes	DB000ZZ, DB001ZZ, DB002ZZ, DB003Z0, DB003ZZ, DB004ZZ, DB005ZZ, DB006ZZ, DB010ZZ, DB011ZZ, DB012ZZ, DB013Z0, DB013ZZ, DB014ZZ, DB015ZZ, DB016ZZ, DB020ZZ, DB021ZZ, DB022ZZ, DB023Z0, DB023ZZ, DB024ZZ, DB025ZZ, DB026ZZ, DB050ZZ, DB051ZZ, DB052ZZ, DB053Z0, DB053ZZ, DB054ZZ, DB055ZZ, DB056ZZ, DB060ZZ, DB061ZZ, DB062ZZ, DB063Z0, DB063ZZ, DB064ZZ, DB065ZZ, DB066ZZ, DB070ZZ, DB071ZZ, DB072ZZ, DB073Z0, DB073ZZ, DB074ZZ, DB075ZZ, DB076ZZ, DB080ZZ, DB081ZZ, DB082ZZ, DB083Z0, DB083ZZ, DB084ZZ, DB085ZZ, DB086ZZ
**Proton Therapy**	CPT/HCPCS Codes	77520,77522,77523,77525
**Surgery**		
**Lobectomy**	CPT/HCPCS Codes	32480, 32663
	ICD-9 Procedure Codes	32.4, 32.41, 32.49
	ICD-10 Procedure Codes	0BTC0ZZ, 0BTC4ZZ, 0BTD0ZZ, 0BTD4ZZ, 0BTF0ZZ, 0BTF4ZZ, 0BTG0ZZ, 0BTG4ZZ, 0BTH0ZZ, 0BTH4ZZ, 0BTJ0ZZ, 0BTJ4ZZ
**Pneumonectomy**	CPT/HCPCS Codes	32440, 32442, 32445, 32488
	ICD-9 Procedure Codes	32.5, 32.50, 32.59
	ICD-10 Procedure Codes	0BTK4ZZ, 0BTL4ZZ, 0BTM4ZZ, 0BTK0ZZ, 0BTL0ZZ, 0BTM0ZZ
**Sublobar Resection**	CPT/HCPCS Codes	32484, 32669, 32505, 32666, 32500[d], 32657[d]
	ICD-9 Procedure Codes	32.20, 32.29, 32.3, 32.30, 32.39

© 2026 Lynch OF et al. *JAMA Network Open.*

	ICD-10 Procedure Codes	0BBC0ZZ, 0BBC4ZZ, 0BBD0ZZ, 0BBD4ZZ, 0BBF0ZZ, 0BBF4ZZ,<br>0BBG0ZZ, 0BBG4ZZ, 0BBH0ZZ, 0BBH4ZZ, 0BBJ0ZZ, 0BBJ4ZZ,<br>0BBK0ZZ, 0BBK4ZZ, 0BBL0ZZ, 0BBL4ZZ, 0BBM0ZZ, 0BBM4ZZ
**Other (e.g., wedge resection or sleeve resection)**	CPT/HCPCS Codes	32482, 32670, 32503, 32504, 32506, 32507, 32667, 32668, 32486
	ICD-9 Procedure Codes	32.9
	ICD-10 Procedure Codes	0B5K0ZZ, 0B5K3ZZ, 0B5K7ZZ, 0B5L0ZZ, 0B5L3ZZ, 0B5L7ZZ, 0B5M0ZZ, 0B5M3ZZ, 0B5M7ZZ, 0BBM3ZZ, 0BBM7ZZ

a Deleted code, effective Jan. 1, 2007
b Deleted code, effective Jan. 1, 2009
c Deleted code, effective Jan. 1, 2012
d Deleted code, effective Jan. 1, 2015

© 2026 Lynch OF et al. *JAMA Network Open.*

eTable 2. SEER Regions – 2008 Study vs Our Study

2008 Study SEER Regions	Current Study SEER Regions
San Francisco-Oakland SMSA (1975+)	San Francisco-Oakland SMSA (1975+)
Connecticut (1975+)	Connecticut (1975+)
Detroit (1975+)	Detroit (1975+)
Hawaii (1975+)	Hawaii (1975+)
Iowa (1975+)	Iowa (1975+)
New Mexico (1975+)	New Mexico (1975+)
Seattle (Puget Sound) (1975+)	Seattle (Puget Sound) (1975+)
Utah (1975+)	Utah (1975+)
Metropolitan Atlanta (1975+)	Metropolitan Atlanta (1975+)
	San Jose-Monterey (1992+)
	Los Angeles (1992+)
	Rural Georgia (1992+)
	Greater California (excl. SF, Los Ang. & SJ) (2000+)
	Kentucky (2000+)
	Louisiana (2000+)
	New Jersey (2000+)
	Greater Georgia (excluding Atlanta and Rural GA)

© 2026 Lynch OF et al. *JAMA Network Open.*

eFigure. Study Selection Criteria

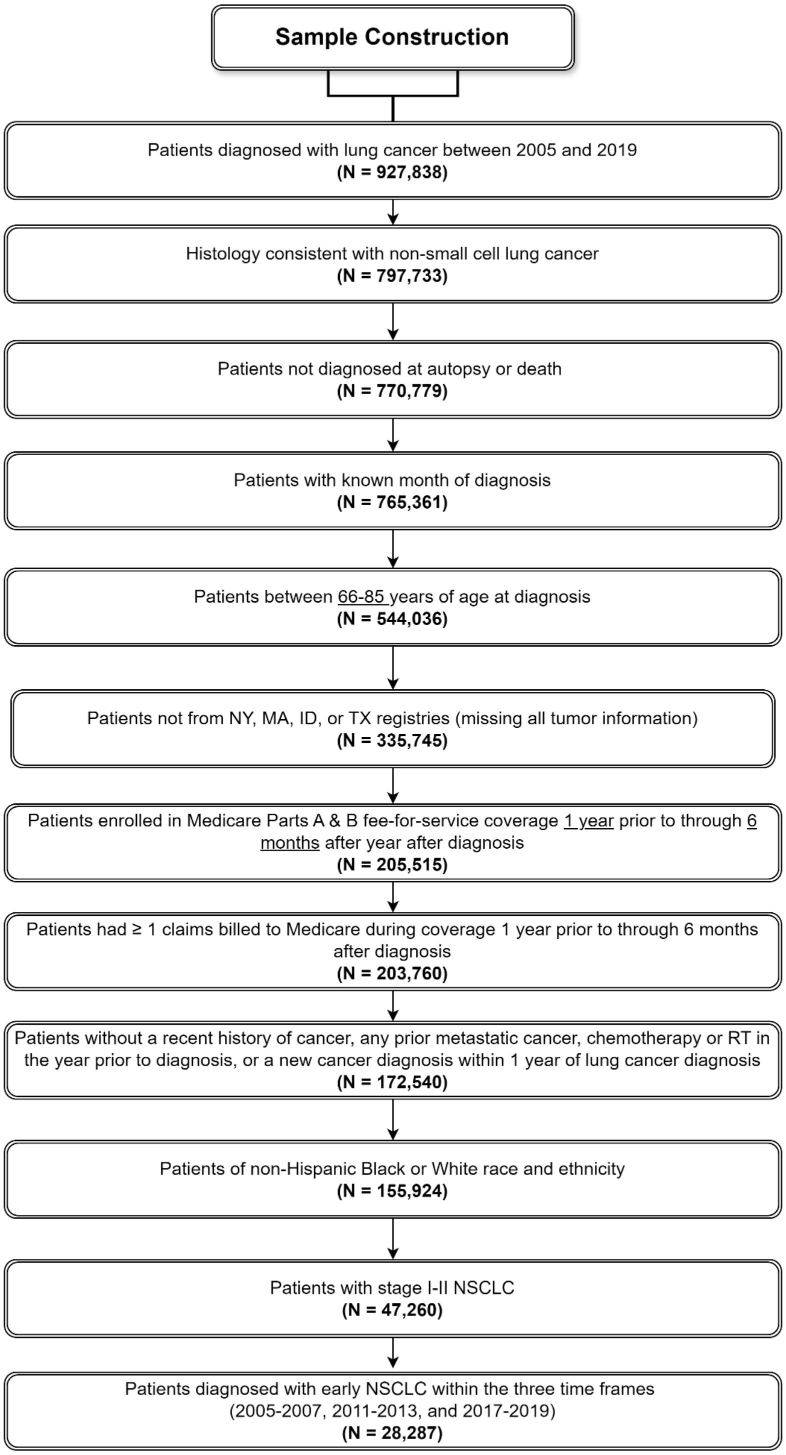

© 2026 Lynch OF et al. *JAMA Network Open.*
