# Supplementary material for: Early-Stage Lung Cancer Treatment Disparities by Race Among Medicare Beneficiaries
Source: JAMA Netw Open. 2026 Mar 2;9(3):e2559845. doi: 10.1001/jamanetworkopen.2025.59845 (PMC12954539; doi:10.1001/jamanetworkopen.2025.59845)
Supplement: Supplement 2. — Data Sharing Statement [file jamanetwopen-e2559845-s002.pdf]

Data Sharing Statement

Lynch. Early-Stage Lung Cancer Treatment Disparities by Race Among Medicare Beneficiaries. *JAMA Netw Open*. Published March 02, 2026. doi:10.1001/jamanetworkopen.2025.59845

Data
Data available: No
